# Relationship of Nitrogen Use Efficiency with the Activities of Enzymes Involved in Nitrogen Uptake and Assimilation of Finger Millet Genotypes Grown under Different Nitrogen Inputs

**DOI:** 10.1100/2012/625731

**Published:** 2012-08-01

**Authors:** Nidhi Gupta, Atul K. Gupta, Vikram S. Gaur, Anil Kumar

**Affiliations:** Department of Molecular Biology and Genetic Engineering, College of Basic Sciences and Humanities, G.B. Pant University of Agriculture and Technology, Uttarakhand Pantnagar 263 145, India

## Abstract

Nitrogen responsiveness of three-finger millet genotypes (differing in their seed coat colour) PRM-1 (brown), PRM-701 (golden), and PRM-801 (white) grown under different nitrogen doses was determined by analyzing the growth, yield parameters and activities of nitrate reductase (NR), glutamine synthetase (GS), glutamate synthase; GOGAT, and glutamate dehydrogenase (GDH) at different developmental stages. High nitrogen use efficiency and nitrogen utilization efficiency were observed in PRM-1 genotype, whereas high nitrogen uptake efficiency was observed in PRM-801 genotype. At grain filling nitrogen uptake efficiency in PRM-1 negatively correlated with NR, GS, GOGAT activities whereas it was positively correlated in PRM-701 and PRM-801, however, GDH showed a negative correlation. Growth and yield parameters indicated that PRM-1 responds well at high nitrogen conditions while PRM-701 and PRM-801 respond well at normal and low nitrogen conditions respectively. The study indicates that PRM-1 is high nitrogen responsive and has high nitrogen use efficiency, whereas golden PRM-701 and white PRM-801 are low nitrogen responsive genotypes and have low nitrogen use efficiency. However, the crude grain protein content was higher in PRM-801 genotype followed by PRM-701 and PRM-1, indicating negative correlation of nitrogen use efficiency with source to sink relationship in terms of seed protein content.

## 1. Introduction

Cereal grains are considered to be one of the most important sources of dietary proteins, carbohydrates, vitamins, minerals, and fiber for people all over the world. Finger millet commonly referred as ragi or mandua ranks fourth in importance among millets in the world after sorghum (*Sorghum bicolor*), pearl millet (*Pennisetum glaucum*), and foxtail millet (**Setaria** *italica*). Finger millet, *Eleusine coracana *(L.) Gaertn subsp. *coracana*, belongs to family Poaceae, subfamily Chloridoideae, and is considered to be a native crop of Central Africa [1]. Finger millet is grown mainly by subsistence farmers and serves as a food security crop [2] because of its high nutritional value and excellent storage qualities. Since finger millet capitalizes on low nitrogen inputs, it could be considered as high nitrogen efficient crop. Thus, it is quite pertinent and promising to study the biochemical mechanism(s) associated with nitrogen use efficiency using this crop as model system.

Nitrogen use efficiency (NUE) at the plant level is its ability to utilize the available nitrogen (N) resources to optimize its productivity. This includes nitrogen uptake and assimilatory processes, redistribution within the cell and balance between storage and current use at the cellular and whole plant level [3, 4]. Nitrogen use efficiency (NUE) for crop plants is of great concerns throughout the world. Burgeoning population of world needs crop genotypes responding to higher nitrogen and showing direct relationship to yield with use of nitrogen inputs, that is, high nitrogen responsive genotypes. However, for fulfilling the high global demand of organic produce, it requires the low nitrogen responsive genotypes with greater NUE and grain yields. Nitrogen use efficiency in plants is a complex quantitative trait that involves many genes and depends on a number of internal and external factors in addition to soil nitrogen availability, such as photosynthetic carbon fixation to provide precursors required for amino acid biosynthesis or respiration to provide energy. The assimilation of inorganic nitrogen into organic form has marked effect on plant productivity, biomass, and crop yield [5, 6]. In all higher plants, inorganic nitrogen is first reduced to ammonia prior to incorporation into organic form [7]. Reduction of nitrate occurs in two distinct reactions catalyzed by different enzymes. The first reaction occurs in cytosol catalyzed by nitrate reductase, which reduces nitrate to nitrite [8]. Nitrite arising in cytosol from nitrate reductase action is transported into chloroplasts in leaves where nitrite is further reduced by the action of nitrite reductase to ammonium ions [7]. Ammonia is assimilated into organic form as glutamine and glutamate, which serves as the nitrogen donors in the biosynthesis of essentially all amino acids, nucleic acids, and other nitrogen containing compounds such as chlorophyll. The individual isoenzymes of glutamine synthetase (GS, E.C.6.3.1.2), glutamate synthase (NADH-GOGAT-E.C.1.4.1.14, FD-GOGAT-E.C.1.4.7.1), and glutamate dehydrogenase (NADH-GDH: EC.1.4.1.2; NADPH-GDH: E.C.1.4.1.4) have been proposed to play important role in three major ammonium assimilation processes: primary nitrogen assimilation, reassimilation of photorespiratory ammonia, and reassimilation of “recycled” nitrogen [7]. Glutamine and glutamate can then be used to form aspartate and asparagines, and these four amino acids are used to translocate organic nitrogen from sources to sinks [9, 10]. The enzymes involved in the primary assimilation of ammonium into these four N-transport amino acids Glu/Gln and Asp/Asn are glutamine synthetase (GS), glutamate synthase (GOGAT), aspartate amino transferase (AAT), and asparagines synthetase (AS). The importance of glutamate dehydrogenase (GDH) in higher plant N metabolism is still controversial, as it has never been clearly demonstrated that the enzyme plays a significant role either in ammonia assimilation or carbon recycling in plants [11, 12]. Moreover, the role of GDH in N management and recycling has recently been reviewed in a number of whole-plant physiological studies performed on tobacco [12] and maize [13].

Since, from both economical and ecological point of view, agricultural practices are going towards extensive systems using lower N fertilizers, a better knowledge of physiological basis of nitrogen use efficiency (NUE) in economically important crop such as finger millet is required. Although finger millet is highly nitrogen use efficient crop yet, there is a wide variation across the genotypic level. Thus, the development of finger millet that can make the best use of N in low-nitrogen soils is essential for the sustainability of agriculture [14]. This highly complex objective requires a deep understanding of the physiological and biochemical responses of finger millet genotypes to different nitrogen levels. In the present investigation, attempts were made to understand the mechanisms associated with NUE and the nitrogen uptake and assimilatory enzymes in finger millet grown under different nitrogen conditions.

## 2. Material and Methods

### 2.1. Plant Material and Nitrogen Treatments

Three finger millet (*Eleusine coracana*) genotypes (differing in their seed coat colour) PRM-1 (brown), PRM-701 (golden), and PRM-801 (white) were grown in pot conditions. For each finger millet variety, four nitrogen treatments were given, namely, high nitrogen dose (60 kg/ha), normal nitrogen dose (40 kg/ha), low nitrogen treatments (20 kg/ha) and farmyard, FYM (7.5 tonnes/hectare) along with control (no nitrogen added). Thus, five soil conditions were used. Nitrogen was applied through urea at three intervals, namely, 50% at the time of sowing, 25% at five leaf stage (30 days after sowing), 25% at the time of flowering/post anthesis. All the pots and control received a basal dose of 20 kg/ha of each muriate of potash and single superphosphates.

### 2.2. Growth Parameters

The plant height and leaf area (LICOR-3000 leaf area meter) were measured at the vegetative stage (40 days after sowing) and the flowering stage. SPAD value was noted by chlorophyll meter SPAD-502 at vegetative stage (40 days after sowing), the flowering stage, and grain filling stages. The dry matter and grain yield were noted at the time of harvest. Heading date was determined by counting the number of days from sowing to 50% of spikes fully emerged from the boot.

### 2.3. Extraction and Assay of Nitrogen Uptake and Ammonium Assimilation Enzymes

The four enzymes, namely, NR, GS, GOGAT, and GDH, were assayed in freshly harvested flag leaf at three different developmental stages of finger millet genotypes. The protein was determined from all of the enzyme extracts [15]. All the assays were done with three replications. Specific activity of an enzyme has been defined as *μ*mol of product formed per mg protein.

#### 2.3.1. Nitrate Reductase (NR)

The nitrate reductase (NR) activity was estimated by using the method described by Hageman and Hucklesby, 1971 [16]. 500 mg of freshly harvested flag leaf tissue were cut into small pieces and were transferred into test tubes containing 3 mL of each 0.2 M potassium phosphate buffer (pH 7.5), and 0.4 M potassium nitratewhich were then incubated in dark at 33°C for 30 min. Add 0.2 mL of above extract after incubation in separate test tube containing 1 mL distilled water. Add 1.2 mL (1 : 1 v/v) mixture of NED (0.1% w/v) and sulphanilamide (1% (w/v) in 3 N HCl) and keep in darkness for 15 min for pink colour development. The absorbance was measured at 540 nm with the help of spectrophotometer using distilled water as blank, and the amount of nitrite present was found out by comparing with the standard curve.

#### 2.3.2. Glutamine Synthetase (GS)

 The extraction buffer included, 10 mM-Tris HCl (pH 7.6), 1 mM-MgCl2, 1 mM-EDTA, and 1 mM-2 mercaptoethanol. Leaves (2 g) were grinded using liquid N2 in the presence of cover slips followed by centrifugation at 12,000 xg for 30 min at 4°C [17]. Supernatant was collected and stored at −20°C. The assays were carried out by continuous spectrophotometric rate determination method.

#### 2.3.3. Glutamate Synthase (GOGAT)

Activity of GOGAT was determined in enzyme preparation described for GS. Standard assay mixture contained 40 mM potassium phosphate buffer (pH 7.5), 10 mM L-glutamine, 10 mM 2-oxoglutarate, 0.14 mM NADH, and crude enzyme (final volume 3 m1). Increase in absorbance at 340 nm for 3-4 min at room temperature (25°C) was recorded. Absorbance (340 nm/min) was calculated from initial linear portion of the curve.

#### 2.3.4. Glutamate Dehydrogenase (GDH)

 Extraction buffer (pH 7.9) consisted of 0.05 M imidazole, 5 mM DTT. Leaves (1 g) were grinded using liquid N2 in the presence of cover slips in chilled mortar and pestle and were centrifuged at 12,000 xg for 40 min at 4°C. Supernatant was collected and stored at −20°C. The assays were carried out by continuous spectrophotometric rate determination method.

### 2.4. Crude Grain Protein Content and Nitrogen Use Efficiency Components

Nitrogen content in grains and straw was determined by micro-Kjeldhal method [18]. The nitrogen content of grain was then multiplied by the factor 6.25 to obtain crude grain protein content and expressed in g per 100 g of grain on a moisture free basis. The following nitrogen efficiency parameters were calculated for each treatment: nitrogen use efficiency, NUE (g g^−1^) as the ratio of grain yield to nitrogen supply, where N supply is the sum of soil NO3^−^-N at planting, mineralized N and N fertilizer; nitrogen utilization efficiency, NU_t_E (g g^−1^) as the ratio of grain yield to total plant nitrogen uptake; nitrogen uptake efficiency, NU_p_E (g g^−1^) as the ratio of total plant N uptake to nitrogen supply.

### 2.5. Grain Weight per Plant

Random sample of the grains from individual genotypes was obtained. These grain samples were dried at room temperature (30°C) to minimize intrinsic moisture content uniformity. Then, these dried grain samples were weighed by electronic weighing balance to detect grain weight per plant.

### 2.6. Statistical Analysis

A complete factorial arrangement of treatments was used (soil condition × genotype) as a complete randomized design with three replications. Mean ± standard error mean (SEM) and critical difference at 5% (CD at 5%) values were calculated for statistical analysis. Correlation coefficients were also measured for various physiological and biochemical parameters.

## 3. Results and Discussion

**Figure 1:**
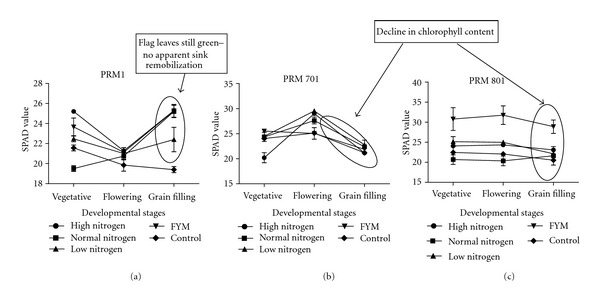
Influence of nitrogen fertilization on SPAD value at various developmental stages of finger millet genotypes (a) brown PRM-1, (b) golden PRM-701, (c) white PRM-801.

There was variation in the heading dates within these finger millet genotypes, that is, the heading date for brown (PRM-1) genotype ranged from 77 to 85 days, whereas for golden (PRM-701) and white (PRM-801) genotypes ranged from 119 to 130 days. This indicates that brown (PRM-1) genotype is early flowering and golden (PRM-701) and white (PRM-801) are late flowering genotypes. Nitrogen fertilization significantly increased plant height and leaf area (Table 1), although there were found differences among the genotypes under different soil conditions. It was observed that, in brown genotype (Figure 1(a)), the SPAD value was higher in vegetative stage when the nitrogen was efficiently taken from the soil and then decreased in flowering and then after third dose of nitrogen there was increment in it. This means that brown genotype might be high nitrogen responsive, whereas, in golden (Figure 1(b)) and white genotypes, (Figure 1(c)), SPAD value was lower in vegetative stage and then increase in flowering, and, then, after third dose of nitrogen, there was decline in it. This means that golden and white genotypes are not able to take nitrogen immediately after addition of nitrogen, that is, they are low nitrogen responsive genotypes. The SPAD readings are calibrated to obtain the chlorophyll content of the leaves or correlated directly with plant performance [19–21], providing a practical method of assessing N status and N requirements. Successful use of chlorophyll meters varies with crop type and has been affected by many factors including varietal differences [22, 23], growth stages [24], nutrient deficiencies other than N [25], environmental conditions [19], and measurement positions on leaves [26]. 1000 grain weight was significantly highest (3.63 g) in white genotype (PRM-801) under low nitrogen condition, and lowest 1000 grain weight (2.09 g) was found in golden genotype under control condition. In the present study, increasing nitrogen rate improved yield attributes and grain yield. The positive effect of the, application of inorganic fertilizers on crop yields, and yield improvements have been reported earlier [27].

Nitrogen use efficiency and nitrogen utilization efficiency were significantly affected by different genotypes and soil conditions. The interaction between genotype and soil condition for nitrogen use efficiency and nitrogen utilization efficiency was significant. However, this was in contrast to nitrogen uptake efficiency. The highest nitrogen use efficiency was observed in brown (PRM-1) genotype (5.1 g g^−1^) followed by golden (PRM-701) genotype (4.1 g g^−1^) and white (PRM-801) genotype (2.4 g g^−1^) relative to control. Similarly, the highest nitrogen utilization efficiency was observed in brown (PRM-1) genotype (2.7 g g^−1^) followed by golden genotype (PRM-701) (2.2 g g^−1^) and white genotype (PRM-801) (1.2 g g^−1^) relative to control. However, the highest nitrogen uptake efficiency was observed in white genotype (PRM-801) (3.0 g g^−1^) followed by golden genotype (PRM-701) (2.7 g g^−1^) and brown genotype (PRM-1) (2.2 g g^−1^) with respect to control. The highest nitrogen use efficiency was observed (Table 2) in brown (PRM-1) genotype (6.67 g g^−1^) under high nitrogen condition, and lowest nitrogen use efficiency (2.40 g g^−1^) was observed in white (PRM-801) genotype under control condition. The highest nitrogen utilization efficiency was observed in brown (PRM-1) genotype (3.61 g g^−1^) under normal nitrogen condition, and lowest nitrogen utilization efficiency (1.17 g g^−1^) in white genotype under control condition. The highest nitrogen uptake efficiency (3.10 g g^−1^) was observed in white (PRM-801) genotype under low nitrogen condition and lowest nitrogen uptake efficiency (2.00 g g^−1^) was observed in brown (PRM-1) under normal nitrogen condition. This indicates that nitrogen use efficiency is positively correlated with nitrogen utilization efficiency which is negatively correlated with nitrogen uptake efficiency. There was positive correlation between nitrogen use efficiency, nitrogen utilization efficiency, and nitrogen uptake efficiency with the yield. Also, the correlation coefficient between nitrogen uptake efficiency and yield (*r* = 0.402) is higher as compared to nitrogen use efficiency (*r* = 0.117) and nitrogen utilization efficiency (*r* = 0.014). As for other cereals, significant differences were obtained for N uptake and efficiency of use in different rice genotypes, N uptake being one of the most important factors controlling yield [28]. Crude grain protein content was highest in white (PRM-801) genotype (9.6%) and lowest in brown (PRM-1) genotype (7.5%) relative to control. It was found that grain protein content, an important grain quality trait, was found to be negatively correlated with nitrogen use efficiency and nitrogen utilization efficiency but positively correlated with nitrogen uptake efficiency. As recently reviewed by Rathke et al. [29], it is clear that to improve seed yield, oil content, and N efficiency in crops like winter oilseed rape the use of N-efficient management strategies is required, including the choice of variety and the form and timing of N fertilization adapted to the site of application.

**Figure 2:**
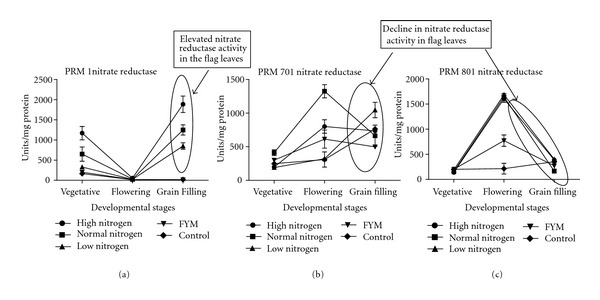
Influence of nitrogen fertilization on nitrate reductase (NR) enzyme activity (specific activity) at various developmental stages of finger millet genotypes (a) brown PRM-1, (b) golden PRM-701, (c) white PRM-801.

From the Figure 2, it was inferred that, after addition of third split dose at flowering, the nitrate reductase activity observed at grain filling was found to be least in white genotype (295 Units/mg protein) followed by golden genotype (736.6 Units/mg protein) and highest in brown genotype (1188.6) indicating that nitrate reductase activity was increased and thus brown is high nitrogen use efficient genotype as compared to other two. The variation in nitrate reductase activity between these genotypes may be associated with difference in regulation of N transporter genes or N fluxes in roots [30]. Roots possess at least three, kinetically distinct, NO^−3^ transport systems [31]. The high affinity transport system(s) mediates most of the uptake activity when the N concentration is lower than 1 mM and the low affinity transport system is responsible for the main uptake when the N concentration is increased above 1 mM [32–34]. This assumption is based on Km value data of rice and Arabidopsis nitrate transporters, because at present the Km value of nitrate transporters of finger millet has not been calculated and reported. However, it could be speculated that the activity of the finger millet transporter may be similar to the rice nitrate transporters. Thus, in brown genotype, there may be low affinity transporters more active in the roots and the shoots, which might be responsible for the higher nitrate reductase activity in flag leaves under high nitrogen conditions after the addition of third dose. However, in golden and white genotypes, high affinity transport system might be more active. Such adaptive regulatory control mechanisms allowing a response to a shortage in N availability may, under certain conditions, be directly controlled through the activity of the nitrate transport system itself, in a given environment [35]. Correlation studies indicates that, at grain filling stage, in brown genotype, nitrogen uptake efficiency has negative correlation with nitrate reductase activity, whereas, in golden and white genotype, nitrogen uptake efficiency has positive correlation with nitrate reductase activity.

**Figure 3:**
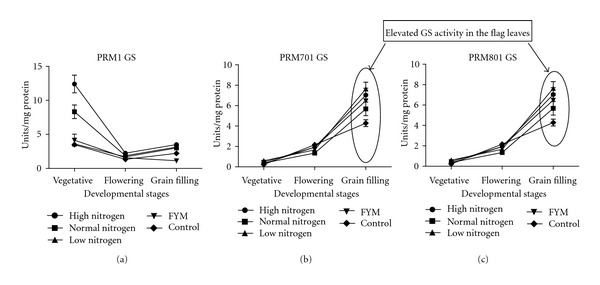
Influence of nitrogen fertilization on glutamine synthetase (GS) enzyme activity (specific activity) at various developmental stages of finger millet genotypes (a) brown PRM-1, (b) golden PRM-701, (c) white PRM-801.

**Figure 4:**
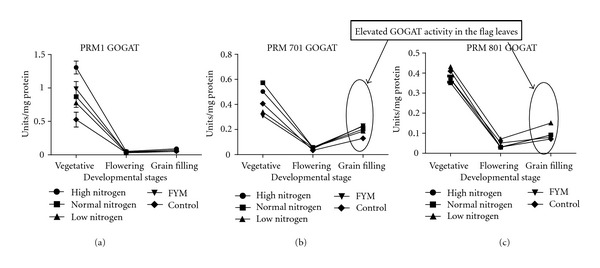
Influence of nitrogen fertilization on glutamate synthase (GOGAT) enzyme activity (specific activity) at various developmental stages of finger millet genotypes (a) brown PRM-1, (b) golden PRM-701, (c) white PRM-801.

**Figure 5:**
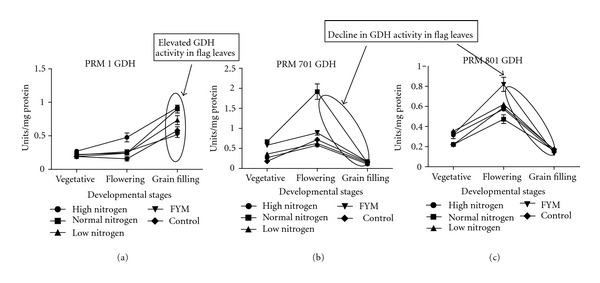
Influence of nitrogen fertilization on glutamate dehydrogenase (GDH) activity (specific activity) at various developmental stages of finger millet genotypes (a) brown PRM-1, (b) golden PRM-701, (c) white PRM-801.

The study of ammonium assimilating enzymes (Figures 3, 4, and 5 **)** at grain filling stage, is important because, at grain filling stage, the N assimilated during the active growth stage is remobilized. The primary pathway for conversion of ammonium into amino acids involves GS and GOGAT [7]. Since the NADH GOGAT isoform is reported to be responsible for grain protein content, its activity in the flag leaves was studied. It has been shown that several rice transgenic lines overproducing NADH-GOGAT showed an increase in grain weight (up to 80%), indicating that this enzyme is indeed a key step for nitrogen reutilization (Yamaya et al., 2002). The GS enzyme activity at grain filling stage was highest for golden genotype (7.67 Units/mg protein) followed by white (5.61 Units/mg protein) and lowest for brown genotype (2.60 Units/mg protein). Similar is the case for GOGAT, that is, the GOGAT enzyme activity at grain filling stage was highest for golden genotype (0.19 Units/mg protein) followed by white (0.10 Units/mg protein) and lowest for brown genotype (0.07 Units/mg protein). Correlation studies indicate that, in brown genotype at grain filling stage, there is positive correlation between nitrogen use efficiency and nitrogen utilization efficiency with GS but GS has negative correlation with nitrogen uptake efficiency. In golden genotype at grain filling, nitrogen use efficiency and nitrogen uptake efficiency have positive correlation with GS, but nitrogen utilization efficiency has negative correlation with GS. In white genotype at grain filling, nitrogen use efficiency, nitrogen utilization efficiency, and nitrogen uptake efficiency have positive correlation with GS. Correlation studies with another ammonium assimilating enzyme, GOGAT in brown genotype at grain filling, indicate that, in brown genotype nitrogen use, nitrogen utilization and nitrogen uptake efficiency have negative correlation with GOGAT. In golden genotype at grain filling, nitrogen use, nitrogen utilization and nitrogen uptake efficiency have positive correlation with GOGAT. In white genotype at grain filling, nitrogen utilization, and nitrogen uptake efficiency have positive correlation with GOGAT except nitrogen use efficiency. This revealed that ammonium assimilation was lowest in brown genotype, and, thus, it correlates with the lowest grain protein content, but, since brown genotype showed highest nitrate reductase activity at grain filling stage, this is compensated by the increased level of dry matter accumulation in it as compared to other two genotypes. To further understand, the rate of nitrogen remobilization glutamate dehydrogenase (GDH) activity at grain filling stage was analysed. The activity of GDH has always been subjected to much controversy. Some researchers report the activity of GDH in senescing leaves the deaminating direction [36, 37] while some report its activity in the aminating direction [38]. Nitrogen assimilation and recycling in young leaves mainly take place within the chloroplast where nitrite reduction occurs and ammonium is assimilated by the GS/GOGAT cycle involving chloroplastic GS2 and Fd-GOGAT. Chloroplast breakdown during senescence involves *de facto* NiR, GS2, and GOGAT proteolysis. In senescing leaves, nitrogen recycling and reassimilation need then to be catalysed by enzymes other than chloroplastic ones. It has been proposed that glutamine is mainly synthesized in senescing leaves by newly expressed GS1 isoforms. Using the amino acid pool released via the proteolysis of chloroplast proteins, a series of transamination reactions leads to an increase in the glutamate pool that could serve immediately as a substrate for GDH, deaminating activity thus providing 2-oxoglutarate and ammonia. Ammonia released this way is in turn reassimilated by GS1 to produce glutamine for export to the developing sink organs [39].

In the present study, GDH activity was found to be highest in brown genotype (0.73 Units/mg protein) (Figure 5) followed by white (0.15 Units/mg protein) and golden genotype (0.14 Units/mg protein). Correlation studies with this another alternative ammonium assimilating enzyme indicate that, in brown PRM-1 genotype nitrogen use, nitrogen utilization and nitrogen uptake efficiency have positive correlation with GDH, whereas, in golden PRM-701 and white PRM-801 genotypes nitrogen use, nitrogen uptake efficiency has negative correlation with GDH. This indicates that redistribution of nitrogen by GDH activity is more in brown PRM-1 genotype as compared to other two genotypes which is compensated by higher dry matter accumulation in brown PRM-1 genotype. Furthermore, according to the data presented here, the activity of GDH appears to be in the deaminating direction. At the maturity stage, the photosynthesis virtually declines because of the hydrolysis of flag leaf cellular components/proteins into transport compounds with low C/N ratio to develop seed for their accumulation. According to the Figure 1, it can be seen that in both the high protein content genotypes “white” and “golden” this hydrolysis was relatively quicker than the low grain protein content brown genotype. That means that at grain filling stage the flag leaves of the brown genotype were much greener than the other two genotypes. Probably, the sequential increase in the activity of GDH and later GS during flowering and during grain filling stages, respectively, in white and golden genotypes quickly transported the nitrogen from early stages of grain filling which continued till the end of grain filling. Probably, this might be a reason for the high grain protein content in the grains of white and golden genotypes. Contrastingly, in case of the brown genotype, GDH activity in the flag leaves was found to be increased and it remained high till the grain filling stage. However, this increase in the GDH activity in the flag leaves was not substantiated with a parallel increase of GS in the flag leaves during grain filling which interestingly coincided with low protein content in the grains. It indicates that both high GS activity along with high GDH activity is probably necessary at the time of flowering to grain filling to carry out the deaminating and transaminating reactions during hydrolysis of flag leaves chloroplast initiated at the time of flowering. It also appears that not only the high activity of GDH but also high activity of GS in the flag leaves at the time of grain filling is necessary to achieve high protein content in the grains. However, further research is needed in this area. Furthermore, there was a direct relationship of grain protein content and GS activity, that is, high grain protein content genotypes had high GS activity in flag leaves. Furthermore, the correlation studies of nitrogen metabolism enzymes at grain filling stage indicate that in brown PRM-1 genotype nitrogen uptake efficiency is negatively correlated with nitrate reductase, glutamine synthetase, glutamate synthase, whereas in golden PRM-701 and white PRM-801 genotype nitrogen uptake efficiency is positively correlated with nitrate reductase, glutamine synthetase, glutamate synthase.

 Therefore, brown PRM-1 genotype is high nitrogen responsive and has high nitrogen use efficiency, whereas golden PRM-701 and white PRM-801 are low nitrogen responsive genotypes and have low nitrogen use efficiency. However, the crude grain protein content was higher in white PRM-801 genotype followed by golden PRM-701 and brown PRM-1 genotypes. From this study, it is inferred that study of biochemical parameter with physiological parameter will enable us to identify genotypes that are beneficial from the agricultural point of view.

## Figures and Tables

**Table 1:** Influence of genotype and soil condition on growth parameters.

	Plant height (cm)		Leaf area (cm^2^)	
	Vegetative	Flowering	Vegetative	Flowering
Brown (PRM-1)				
High nitrogen	11	78.25	16.75	28
Normal nitrogen	9.1	57.4	13.3	26.3
Low nitrogen	7.6	56.5	12.03	24.1
FYM	8.8	78.73	15.1	29
Control	8.2	41.7	9.4	24

Golden (PRM-701)				
High nitrogen	11.1	73.3	18	27
Normal nitrogen	10.3	77.9	12	36
Low nitrogen	7.7	72.3	16	27.4
FYM	11.6	75	15.3	27.6
Control	9.5	60	10.7	25

White (PRM-801)				
High nitrogen	14.3	68.99	18.6	32.5
Normal nitrogen	13.1	76	14.6	28
Low nitrogen	13.6	70	17	32
FYM	17.6	97	19.7	37.8
Control	11.1	67	13	26

SEm±	1.10	2.35	0.93	1.87
CD at 5%				
Genotype	1.43	3.04	1.19	2.42
Soil condition	1.84	3.93	1.54	3.12
Genotype X soil condition	3.19	6.81	2.67	5.40

**Table 2:** Influence of genotype and soil condition on yield parameters.

	Dry matter (g)	1000 grain weight (g)	Number of grains per spike	Crude grain protein content (g per 100 g of grain)	Nitrogen use efficiency (g g^-1^)	Nitrogen utilization efficiency (g g^-1^)	Nitrogen uptake efficiency (g g^-1^)
Brown (PRM-1)							
High nitrogen	7.0 (180)	2.7 (17.4)	680 (36)	8.1 (8)	6.7 (31.8)	3.4 (25.9)	2.2 (0)
Normal nitrogen	5.8 (132)	2.4 (4.3)	665 (33)	7.6 (1.6)	6.0 (18.1)	3.6 (33.7)	2.0 (−9.09)
Low nitrogen	3.5 (40)	2.7 (17.4)	600 (20	7.9 (4.9)	5.5 (8.9)	3.1 (12.9)	2.2 (0.0)
FYM	5.6 (124)	2.5 (8.7)	570 (14)	8.0 (6.7)	6.4 (25.5)	3.4 (27.0)	2.2 (0.0)
Control	2.5	2.3	500	7.5	5.1	2.7	2.2

Golden (PRM-701)							
High nitrogen	3.3 (65)	3.1 (55.0)	530 (51)	9.1 (1.3)	6.1 (48.3)	3.3 (50.0)	2.4 (−11.1)
Normal nitrogen	3.9 (95)	3.2 (60.0)	480 (37)	9.6 (6.5)	6.0 (45.9)	3.3 (50.0)	2.7 (0.0)
Low nitrogen	3.6 (80)	3.0 (50.0)	600 (71)	9.8 (8.3)	7.4 (81.5)	3.4 (54.5)	2.9 (7.4)
FYM	7.0 (250)	3.0 (50.0)	450 (29)	9.1 (1.1)	4.0 (−3.7)	2.2 (0.0)	2.4 (−11.1)
Control	2.0	2.0	350	9.0	4.1	2.2	2.7

White (PRM-801)							
High nitrogen	2.5 (25)	3.0 (36.4)	420 (40)	9.8 (1.6)	3.1 (29.2)	1.5 (25.0)	2.6 (−13.3)
Normal nitrogen	2.6 (30)	2.8 (27.3)	400 (33)	9.9 (2.9)	4.3 (79.2)	2.1 (75.0)	2.9 (−3.3)
Low nitrogen	4.2 (110)	3.6 (63.6)	370 (23)	10 (4.2)	4.5 (87.5)	1.9 (58.3)	3.1 (3.3)
FYM	6.0 (200)	3.3 (50.0)	360 (20)	10 (4.2)	4.4 (83.3)	2.3 (91.7)	2.7 (−10.0)
Control	2.0	2.2	300	9.6	2.4	1.2	3.0

SEm±	0.46	0.15	37.88	0.35	0.37	0.11	0.17
CD at 5%							
Genotype	0.59	0.18	48.93	0.45	0.47	0.14	0.22
Soil condition	0.76	0.24	63.17	NS	0.61	0.18	NS
Genotype X soil condition	1.31	0.42	109.41	NS	1.06	0.32	NS

Parenthesis: % increase with respect to control of particular genotype.

## Abbreviations

NR: Nitrate reductase
GS: Glutamine synthetase
GOGAT: Glutamate synthase
GDH: Glutamate dehydrogenase
NUE: Nitrogen use efficiency
NU_*t*_E: Nitrogen utilization efficiency
NU_*p*_E: Nitrogen uptake efficiency.
